# Complete Genome Sequence of Bacillus subtilis Strain MH1, Which Has a High Level of Bacteriocin-Like Activity, Isolated from Soil in Bangladesh

**DOI:** 10.1128/genomeA.00516-18

**Published:** 2018-06-21

**Authors:** Mohammad Shahnoor Hossain, Marufa Zerin Akhter, Muhammad Maqsud Hossain, M. Asaduzzaman Shishir, Shakila Nargis Khan, M. Mozammel Hoq

**Affiliations:** a Department of Genetic Engineering & Biotechnology, University of Dhaka, Dhaka, Bangladesh; b Department of Microbiology, University of Dhaka, Dhaka, Bangladesh; c NSU Genome Research Institute (NGRI), North South University, Dhaka, Bangladesh; d Department of Biochemistry and Microbiology, North South University, Dhaka, Bangladesh

## Abstract

Bacillus subtilis MH1 demonstrates a high level of bacteriocin activity against several pathogenic bacteria. We announce here the full-genome sequence of strain MH1, isolated from soil in Bangladesh. This genome length is 4,094,053 bp, with 43.5% GC content, 4,217 coding sequences (CDS), 10 rRNA, 84 tRNA, and 1 transfer-messenger RNA (tmRNA).

## GENOME ANNOUNCEMENT

Bacillus subtilis is a Gram-positive, rod-shaped, endospore-forming bacterium which has immense biotechnological importance. This bacterium is normally present in the upper soil and in the gastrointestinal tract of ruminating animals and humans as normal microbiota (1). B. subtilis strain MH1, which displays strong antagonistic activity against several pathogenic bacteria, was isolated from a soil sample in Bangladesh in our laboratory. Based on morphological, biochemical, and 16S rRNA gene sequencing, we classified it as a new subspecies of B. subtilis.

Complete genome sequencing has an important role in the development of omics approaches to studying particular characteristics of a bacterium, to studying gene expression patterns, and to engineering the genome to produce a new recombinant strain with elevated production capacity or an increased level of efficiency for a particular trait. The genome sequence of B. subtilis MH1 is the first complete genome sequence of a B. subtilis strain to be published from Bangladesh. The genome was sequenced by the Illumina MiSeq technology (Illumina, San Diego, CA, USA) at the Genome Research Institute of North South University (NSU), Bangladesh, after construction of a 300-bp paired-end library. Assembly of the raw genome data was performed using SPAdes version 3.11 (2). The scaffold generated was mapped and ordered using the software ABACAS (3), using the reference genome Bacillus subtilis subsp. subtilis strain 168 (GenBank accession number AL009126).

We used Prokka (4) for structural gene prediction and functional annotation. The genome was automatically annotated by Rapid Annotations using Subsystems Technology (RAST) 2.0 (5). The RNAmmer 1.2 software (6) was used for rRNA and tRNA predictions. The correction of the coding sequence was carried out by the Artemis software (7) linked with the BLASTp (https://blast.ncbi.nlm.nih.gov/Blast.cgi?PAGE=Proteins) and UniProt (http://www.uniprot.org) databases.

The total size of the draft genome was 4,094,053 bp, arranged into 64 contigs after initial assembly. The GC content was 43.5%, with an *N*_50_ of 317,850 bp. After scaffolding using the reference genome, all 64 contigs were mapped against the reference genome, resulting in a single contig. This genome contains 4,217 coding sequences (CDS), 10 rRNA, 84 tRNA, and 1 transfer-messenger RNA (tmRNA). BAGEL (8) and antiSMASH 3.0 (9) revealed two lantibiotic-encoding genes along with regulatory genes, immunity proteins, and transporters in the draft genome.

### Accession number(s).

The draft sequence of the genome has been deposited in GenBank under the accession number PYBK00000000.
